# Ischemic Stroke After Bivalent COVID-19 Vaccination: Self-Controlled Case Series Study

**DOI:** 10.2196/53807

**Published:** 2024-06-25

**Authors:** Stanley Xu, Lina S Sy, Vennis Hong, Kimberly J Holmquist, Lei Qian, Paddy Farrington, Katia J Bruxvoort, Nicola P Klein, Bruce Fireman, Bing Han, Bruno J Lewin

**Affiliations:** 1 Department of Research & Evaluation Southern California Permanente Medical Group Pasadena, CA United States; 2 School of Mathematics and Statistics The Open University United Kingdom; 3 Department of Epidemiology University of Alabama at Birmingham Birmingham, AL United States; 4 Kaiser Permanente Vaccine Study Center Kaiser Permanente Northern California Oakland, CA United States

**Keywords:** ischemic stroke, bivalent COVID-19 vaccine, influenza vaccine, self-controlled case series, coadministration, ischemic, stroke, TIA, transient ischemic attack, ischemia, cardiovascular, COVID-19, SARS-CoV-2, vaccine, vaccines, vaccination, association, correlation, risk, risks, adverse, side effect, subgroup analyses, subgroup analysis, bivalent, influenza, infectious, respiratory, incidence, case series

## Abstract

**Background:**

The potential association between bivalent COVID-19 vaccination and ischemic stroke remains uncertain, despite several studies conducted thus far.

**Objective:**

This study aimed to evaluate the risk of ischemic stroke following bivalent COVID-19 vaccination during the 2022-2023 season.

**Methods:**

A self-controlled case series study was conducted among members aged 12 years and older who experienced ischemic stroke between September 1, 2022, and March 31, 2023, in a large health care system. Ischemic strokes were identified using *International Classification of Diseases, Tenth Revision* codes in emergency departments and inpatient settings. Exposures were Pfizer-BioNTech or Moderna bivalent COVID-19 vaccination. Risk intervals were prespecified as 1-21 days and 1-42 days after bivalent vaccination; all non–risk-interval person-time served as the control interval. The incidence of ischemic stroke was compared in the risk interval and control interval using conditional Poisson regression. We conducted overall and subgroup analyses by age, history of SARS-CoV-2 infection, and coadministration of influenza vaccine. When an elevated risk was detected, we performed a chart review of ischemic strokes and analyzed the risk of chart-confirmed ischemic stroke.

**Results:**

With 4933 ischemic stroke events, we found no increased risk within the 21-day risk interval for the 2 vaccines and by subgroups. However, risk of ischemic stroke was elevated within the 42-day risk interval among individuals aged younger than 65 years with coadministration of Pfizer-BioNTech bivalent and influenza vaccines on the same day; the relative incidence (RI) was 2.13 (95% CI 1.01-4.46). Among those who also had a history of SARS-CoV-2 infection, the RI was 3.94 (95% CI 1.10-14.16). After chart review, the RIs were 2.34 (95% CI 0.97-5.65) and 4.27 (95% CI 0.97-18.85), respectively. Among individuals aged younger than 65 years who received Moderna bivalent vaccine and had a history of SARS-CoV-2 infection, the RI was 2.62 (95% CI 1.13-6.03) before chart review and 2.24 (95% CI 0.78-6.47) after chart review. Stratified analyses by sex did not show a significantly increased risk of ischemic stroke after bivalent vaccination.

**Conclusions:**

While the point estimate for the risk of chart-confirmed ischemic stroke was elevated in a risk interval of 1-42 days among individuals younger than 65 years with coadministration of Pfizer-BioNTech bivalent and influenza vaccines on the same day and among individuals younger than 65 years who received Moderna bivalent vaccine and had a history of SARS-CoV-2 infection, the risk was not statistically significant. The potential association between bivalent vaccination and ischemic stroke in the 1-42–day analysis warrants further investigation among individuals younger than 65 years with influenza vaccine coadministration and prior SARS-CoV-2 infection. Furthermore, the findings on ischemic stroke risk after bivalent COVID-19 vaccination underscore the need to evaluate monovalent COVID-19 vaccine safety during the 2023-2024 season.

## Introduction

On August 31, 2022, the US Food and Drug Administration (FDA) granted emergency use authorizations for the Pfizer-BioNTech bivalent COVID-19 vaccine for individuals aged 12 years and older and the Moderna bivalent COVID-19 vaccine for individuals aged 18 years and older [1,2]. The bivalent vaccines contain messenger ribonucleic acid (mRNA) components derived from both the original strain of SARS-CoV-2 and the Omicron variant BA.4 and BA.5 sublineages. Designed to be administered as a single booster dose, bivalent COVID-19 vaccines were recommended to be given ≥60 days after either the primary vaccination or a monovalent booster dose [2]. In the context of waning protection from primary vaccination, bivalent vaccines enhanced the immune response and boosted protection against the virus, offering an additional layer of defense for previously vaccinated individuals [3-5].

Safety data for bivalent mRNA COVID-19 vaccines were initially limited. Because the chemical components and production processes between monovalent and bivalent vaccines were similar, the FDA granted emergency use authorizations for the bivalent COVID-19 vaccines based on safety data for monovalent vaccines, as well as limited bivalent safety data from clinical trials [1,2]. To monitor safety postlicensure, a study using V-safe and the Vaccine Adverse Event Reporting System (VAERS) examined bivalent booster vaccinations in individuals aged older than or equal to 12 years and found that the safety profile was similar to that described for monovalent booster vaccinations [6]. A recent study that comprehensively assessed potential adverse events associated with bivalent vaccines using TreeScan in the Vaccine Safety Datalink (VSD) network found no increased risk for a broad range of adverse events [7].

The VSD has monitored COVID-19 vaccine safety since vaccinations began in December 2020 [8]. In late 2022, VSD’s rapid cycle analyses detected a safety signal for ischemic stroke following the Pfizer-BioNTech COVID-19 bivalent booster vaccination among those 65 years and older, particularly among those who had received a bivalent booster dose and a high-dose or adjuvanted influenza vaccine on the same day (coadministration) [9]. The US Centers for Disease Control and Prevention (CDC) and FDA announced this safety signal in January 2023 [10]. This safety signal attenuated as data accumulated [11]. Another cohort study among adults aged 65 years and older reported that those who received the Pfizer-BioNTech bivalent booster had a similar hazard for ischemic stroke encounters compared to those who received the Moderna bivalent booster vaccine, but had a lower hazard than those who received the Pfizer-BioNTech or Moderna monovalent boosters [12]. In another study, compared to monovalent vaccination, bivalent vaccination was not found to be associated with an increased risk of ischemic stroke, hemorrhagic stroke, myocardial infarction, and pulmonary embolism [13]. A self-controlled case series (SCCS) study conducted in England showed no indication of an increased risk of ischemic stroke risk within 21 days following administration of either of the 2 mRNA COVID-19 bivalent vaccines. Similar findings were observed for individuals aged 65 years and older who received the influenza vaccine concurrently with the bivalent COVID-19 vaccines [14]. Another SCCS study conducted in Israel also did not find an increased risk of ischemic stroke following monovalent or bivalent mRNA COVID-19 vaccine boosters in at-risk populations [15]. A study of Medicare beneficiaries aged 65 years or older showed no significantly elevated risk for stroke immediately after receiving either COVID-19 bivalent vaccine [16]. However, among beneficiaries who had a stroke after getting either COVID-19 bivalent vaccine along with a high-dose or adjuvanted influenza vaccine, there was a significant association between vaccination and nonhemorrhagic stroke within 22 to 42 days for the Pfizer-BioNTech COVID-19 bivalent vaccine. Additionally, there was a significant association between vaccination and transient ischemic attack within 1 to 21 days for the Moderna COVID-19 bivalent vaccine.

The objective of this study was to assess the risk of ischemic stroke after bivalent COVID-19 vaccination among individuals enrolled in Kaiser Permanente Southern California (KPSC) using a modified SCCS design. Subgroup analyses were also conducted by age (younger than 65 years vs older than or equal to 65 years), history of SARS-CoV-2 infection, and coadministration of influenza vaccine.

## Methods

### Study Population and Study Period

We conducted an SCCS study among members aged 12 years and older from KPSC, a large integrated health care system in the United States. The SCCS method is an alternative to standard epidemiological study designs and has been used for evaluating vaccine safety and in fields such as pharmacoepidemiology [17-21]. In an SCCS study, only individuals who have experienced an event are included. Since individuals serve as their own control and the incidence rates of the outcome of interest are compared within individuals, all time-invariant confounding variables are controlled. The SCCS analytic data sets included individuals who experienced ischemic stroke events between September 1, 2022, and March 31, 2023, had completed a COVID-19 vaccine primary series, and had received their last monovalent dose ≥60 days before September 1, 2022. We required KPSC membership on September 1, 2022.

### Exposure and Observation Period

The exposure was defined as the administration of the Pfizer-BioNTech bivalent COVID-19 vaccine (for individuals aged 12 years and older ) or the Moderna bivalent COVID-19 vaccine (for individuals aged 18 years and older) between September 1, 2022, and March 31, 2023. The observation period for the recipients of a bivalent COVID-19 vaccine started on September 1, 2022, and ended on March 31, 2023, or upon death, receipt of the second bivalent dose, or disenrollment, whichever came first.

To adjust for seasonality, we also included ischemic stroke events occurring among eligible individuals aged 12 years and older who had completed a primary series and had received their last monovalent dose ≥60 days before September 1, 2022, but who did not receive a bivalent vaccine during September 1, 2022, to March 31, 2023 (nonbivalent recipients; NBRs). The observation period for ischemic stroke events among NBR started on September 1, 2022, and ended on March 31, 2023, or upon death or disenrollment, whichever came first.

### Outcome

The outcome was defined as the first occurrence of an ischemic stroke event between September 1, 2022, and March 31, 2023 [22]. Ischemic stroke events were identified through medical encounters with an *International Classification of Diseases, Tenth Revision* (*ICD-10*) diagnosis code of G45.8, G45.9, or I63.x, where *x* represents any additional characters in the *ICD-10* code range for ischemic stroke, in the emergency departments or inpatient settings. We also looked back 30 days prior to September 1, 2022, to ensure that the episode was incident. We excluded ischemic stroke events due to other possible causes and adjusted the onset date (details in Multimedia Appendix 1). We considered these ischemic stroke events that were identified with *ICD-10* codes to be electronically identified ischemic stroke events.

### Covariates

We collected demographic variables (age, sex, and race or ethnicity) and the Charlson Comorbidity Index to describe the characteristics of the study population, as well as concomitant influenza vaccination during the study period and history of SARS-CoV-2 infection in the year prior to September 1, 2022.

### Statistical Analyses

We assessed the risk of ischemic stroke following the administration of the Pfizer-BioNTech and Moderna bivalent COVID-19 vaccines separately. Demographic characteristics of individuals who experienced ischemic stroke events during the study period were described among Pfizer-BioNTech bivalent vaccine recipients, Moderna bivalent vaccine recipients, and NBRs.

The risk intervals were prespecified as 1-21 days and 1-42 days after administration of bivalent COVID-19 vaccines, with person-time outside of these risk intervals serving as the control interval. The risk intervals started on the day of vaccination (day 1). Because individuals who had ischemic stroke events might be likely to postpone or avoid bivalent vaccination, we used a modified SCCS approach for event-dependent exposures [22]. The modified SCCS used a pseudo-likelihood approach in the counterfactual framework to estimate the relative incidence (RI) and 95% CIs of events comparing the risk intervals to their corresponding control intervals by maximizing a Poisson pseudolikelihood [23]. In the SCCS analyses, ischemic stroke events occurring among eligible individuals who did not receive the bivalent vaccines were included to account for seasonality, by incorporating calendar month into the model [22]. Given that age did not significantly vary during the relatively short observation period of 7 months, it was not adjusted as a time-varying covariate.

Additionally, we performed several subgroup analyses based on age (younger than 65 vs 65 years and older), coadministration of bivalent COVID-19 vaccine with same-day influenza vaccine (yes or no), and history of SARS-CoV-2 infection (confirmed by a positive laboratory test or a COVID-19 diagnosis) within 1 year prior to September 1, 2022.

When a safety signal (ie, the lower bound of the 95% CI for RI exceeded 1.0) was detected in analyses of electronically identified ischemic stroke events, we conducted a chart review among recipients of bivalent COVID-19 vaccines to confirm ischemic stroke events and identify onset date to determine whether confirmed ischemic stroke events fell in the risk or control interval; confirmation rates were then calculated (number of confirmed events divided by the number of electronically identified events reviewed). We did not conduct a chart review on ischemic stroke events among NBR due to the large number of events in this group and limited resources. In analyses of confirmed ischemic stroke events among recipients of bivalent COVID-19 vaccines, we introduced a randomized allocation of confirmed case status to the NBR group. This allocation was guided by the confirmation rates observed among recipients of bivalent COVID-19 vaccines, as outlined by Xu et al [24]. A total of 5 simulated data sets were generated to replicate the allocation process. The SCCS analyses were conducted on each data set, and the resulting estimates were aggregated using Rubin’s [25] rule, which accounts for both the variability within individual data sets and the variability across multiple data sets. Attributable risk (AR) was calculated using the approach described in Farrington et al [26]:



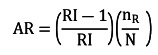



Here, RI is the relative incidence; n_R_ is the number of ischemic stroke events in the risk interval; and N is the number of recipients of a vaccine or dose. The reciprocal of AR is the number needed to harm (NNH). Analyses were conducted using SAS (version 9.4; SAS Institute) and the SCCS models were fitted with the R package (R Core Team) *SCCS* [27].

### Ethical Considerations

Ethics approval for this study was obtained from the KPSC institutional review board on June 6, 2023. In accordance with 45CFR 46.116, informed consent was waived by the institutional review board because the research activities (secondary analyses of electronic health records data) presented no more than minimal risk to participants. Patients or members of the public were not involved in the design, conduct, reporting, or dissemination plans of the research. To protect the privacy and confidentiality of human participants, all staff working on the research study were trained in procedures to protect the privacy of medical record information. All research data were stored behind a firewall in a password-protected network within the Department of Research & Evaluation at KPSC. Study participants were not compensated given the observational nature of the study.

## Results

### Characteristics of Individuals Who Had Ischemic Stroke Events

Table 1 shows the characteristics of individuals who had ischemic stroke events. In total, there were 1057 ischemic stroke events among recipients of the Pfizer-BioNTech bivalent vaccine with a mean length of observation period of 204 (SD 27) days (ranging from 16 to 212 days), 827 ischemic stroke events among recipients of the Moderna bivalent vaccine with a mean length of observation period of 206 (SD 24) days (ranging from 31 to 212 days), and 3049 ischemic stroke events among NBR with a mean length of observation period of 197 (SD 40) days (ranging from 11 to 212 days). Notably, the majority of ischemic stroke events occurred among individuals aged 65 years or older. Those aged younger than 65 years old had fewer comorbidities than those aged 65 years or older (Multimedia Appendix 2).

**Table 1:** Characteristics of individuals who had ischemic stroke events among members of Kaiser Permanente Southern California during the period from September 1, 2022, to March 31, 2023.

			Recipients of Pfizer-BioNTech bivalent COVID-19 vaccine (n=1057)		Recipients of Moderna bivalent COVID-19 vaccine (n=827)		Nonrecipients of bivalent vaccines (n=3049)
**Age (years), n (%)**							
	12-17	1 (0.1)		N/A^a^		6 (0.2)	
	18-44	32 (3.0)		15 (1.8)		261 (8.6)	
	45-64	245 (23.2)		177 (21.4)		1011 (33.2)	
	65-74	618 (58.5)		501 (60.6)		1406 (46.1)	
	≥75	161 (15.2)		134 (16.2)		365 (12.0)	
**Sex, n (%)**							
	Female	540 (51.1)		446 (53.9)		1637 (53.7)	
	Male	517 (48.9)		381 (46.1)		1412 (46.3)	
**Race or ethnicity, n (%)**							
	Hispanic	311 (29.4)		259 (31.3)		1186 (38.9)	
	Non-Hispanic White	470 (44.5)		326 (39.4)		1027 (33.7)	
	Non-Hispanic Asian	119 (11.3)		99 (12.0)		270 (8.9)	
	Non-Hispanic Black	139 (13.1)		120 (14.5)		472 (15.5)	
	Missing	6 (0.6)		9 (1.1)		37 (1.2)	
	Multiple or other	12 (1.1)		14 (1.7)		57 (1.9)	
Length of observation period in days, mean (SD)			204.3 (26.8)		205.7 (23.5)		196.7 (39.9)
Death, n (%)			50 (4.7)		37 (4.5)		309 (10.1)

^a^N/A: not applicable.

### Risk of Ischemic Stroke Following the Pfizer-BioNTech Bivalent COVID-19 Vaccine

For the Pfizer-BioNTech bivalent COVID-19 vaccine, there were 103 electronically identified ischemic stroke events in the 21-day postvaccination risk interval, 954 events in the control interval, and 3049 events among NBR; the overall RI was 0.90 (95% CI 0.73-1.12; Multimedia Appendix 3). The RI was not significantly above 1 across all subgroup analyses by age, coadministration of influenza vaccine, and history of SARS-CoV-2 infection.

In analyses extending the risk interval to 1-42 days following bivalent vaccination, the overall RI was 0.97 (95% CI 0.81-1.15; Table 2). However, in subgroup analyses using the 1-42–day risk interval, we observed an increased risk of ischemic stroke only among individuals younger than 65 years of age who also received an influenza vaccine on the same day. The RI in this subgroup was 2.13 (95% CI 1.01-4.46). Among the subset who also had a documented history of SARS-CoV-2 infection within the year preceding the study period, the RI increased to 3.94 (95% CI 1.10-14.16).

**Figure 1:**
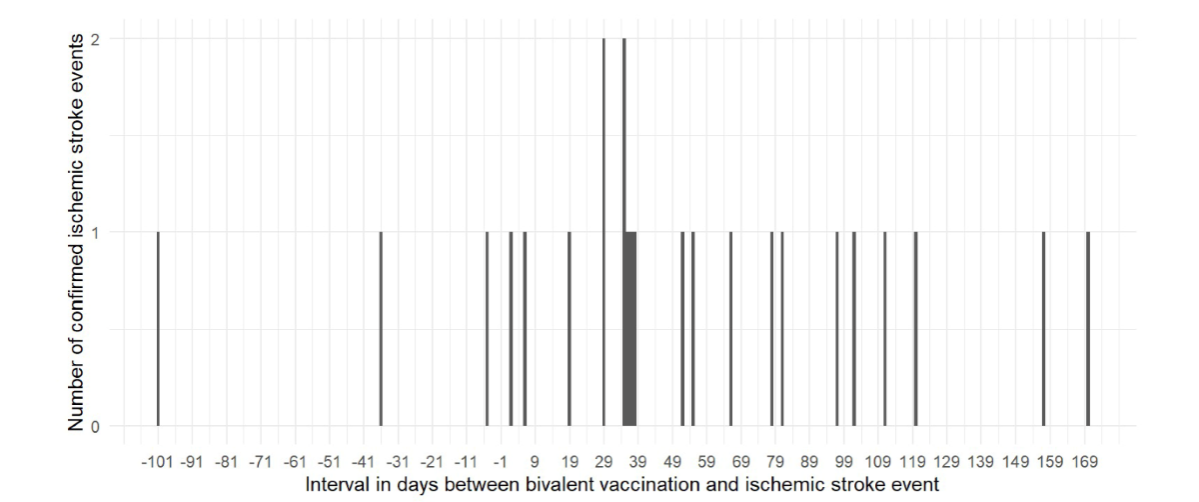
Number of confirmed ischemic stroke events over the interval in days between bivalent vaccination and ischemic stroke event among those who received the Pfizer-BioNTech bivalent vaccine and influenza vaccine on the same day among members of Kaiser Permanente Southern California during the period from September 1, 2022, to March 31, 2023.

In this 1-42–day risk interval analysis of the specific subgroup of individuals aged younger than 65 years who received bivalent and influenza vaccines on the same day, chart review of the 37 electronically identified ischemic stroke events found that 2 were determined to be hemorrhagic strokes and 11 were subsequently found to not meet the criteria for true ischemic stroke events, yielding a confirmation rate of 65% (n=24). With the verified 24 ischemic stroke events and ischemic stroke events among NBR (not verified through chart review, but adjusted for using a 65% confirmation rate), we proceeded to reevaluate the RI in this subgroup. The number of confirmed ischemic stroke events was graphed over the interval in days between bivalent vaccination and ischemic stroke event (Figure 1). There were 10 events in the 1-42–day risk interval and 14 events in the control interval. Using a risk interval of 1-42 days after coadministration of the Pfizer-BioNTech bivalent vaccine and influenza vaccine, the overall RI derived from analyzing confirmed ischemic stroke events among those aged younger than 65 years was 2.34 (95% CI 0.97-5.65; *P*=.06; Table 3). Between September 1, 2022, and March 31, 2023, a total of 117,423 individuals aged younger than 65 years received the Pfizer-BioNTech bivalent vaccine and influenza vaccine on the same day. According to the equation, AR=4.88×10^–5^ and NNH=20,505 with a risk interval of 1-42 days. Among 21,128 individuals who also had a documented history of SARS-CoV-2 infection within the year preceding the study period, the RI increased to 4.27 (95% CI 0.97-18.85; *P*=.06; Table 3); according to the equation, AR=1.45×10^–4^ and NNH=6897 with a risk interval of 1-42 days. Among the 10 confirmed ischemic stroke events in the risk interval of 1-42 days, the mean age was 58 (SD 5) years, ranging from 48 to 63 years. Among these cases, 2 individuals had a documented history of previous ischemic stroke, and no one died as of March 31, 2023. In addition, 7 received the standard dose, egg-based quadrivalent influenza vaccine, while 3 received an influenza vaccine of unknown formulation.

**Table 2:** Numbers of electronically identified ischemic stroke events and relative incidences in the 42 days after Pfizer-BioNTech bivalent COVID-19 vaccination among members of Kaiser Permanente Southern California during the period from September 1, 2022, to March 31, 2023.

		All ages						<65 years old						≥65 years old				
		Number of events, n				Relative incidence (95% CI)		Number of events, n				Relative incidence (95% CI)		Number of events, n				Relative incidence (95% CI)
		Risk interval	Control interval	NBRs^a^			Risk interval		Control interval	NBRs			Risk interval		Control interval	NBRs		
**All recipients**																		
	Overall	212	845	3049	0.97 (0.81-1.15)		50		228	1278	0.98 (0.69-1.38)		162		617	1771	0.96 (0.79-1.17)	
	With history of SARS-CoV-2^b^	35	139	565	0.98 (0.66-1.47)		15		53	292	1.22 (0.64-2.33)		20		86	273	0.83 (0.50-1.40)	
	Without history of SARS-CoV-2	177	706	2484	0.97 (0.80-1.17)		35		175	986	0.91 (0.61-1.38)		142		531	1498	0.99 (0.80-1.22)	
**Coadministration of influenza vaccine**																		
	Overall	41	126	3049	0.97 (0.66-1.41)		15		22	1278	2.13 (1.01-4.46)		26		104	1771	0.75 (0.47-1.19)	
	With history of SARS-CoV-2^b^	9	20	565	1.26 (0.56-2.82)		6		4	292	3.94 (1.10-14.16)		3		16	273	0.51 (0.14-1.90)	
	Without history of SARS-CoV-2	32	106	2484	0.91 (0.59-1.40)		9		18	986	1.57 (0.61-4.05)		23		88	1498	0.80 (0.49-1.31)	
**No coadministration of influenza vaccine**																		
	Overall	171	719	3049	0.96 (0.80-1.17)		35		206	1278	0.81 (0.54-1.20)		136		513	1771	1.01 (0.81-1.25)	
	With history of SARS-CoV-2^b^	26	119	565	0.89 (0.56-1.40)		9		49	292	0.82 (0.37-1.80)		17		70	273	0.88 (0.50-1.55)	
	Without history of SARS-CoV-2	145	600	2484	0.99 (0.80-1.22)		26		157	986	0.82 (0.52-1.29)		119		443	1498	1.04 (0.82-1.31)	

^a^NBR: nonbivalent recipient. These were eligible individuals who did not receive a bivalent vaccine but had completed a primary series of COVID-19 vaccination and had their last monovalent dose ≥60 days before September 1, 2022. Inclusion of these events helps to adjust for temporal trends (seasonality). The same NBR population was used in overall bivalent analyses, as well as bivalent analyses stratified by coadministration of influenza vaccine.

^b^Had SARS-CoV-2 infection (ie, SARS-CoV-2 positive laboratory test or a COVID-19 diagnosis) during the year prior (August 31, 2021-August 31, 2022).

**Table 3:** Numbers of confirmed^a^ ischemic stroke events among recipients of the Pfizer-BioNTech bivalent COVID-19 vaccine aged <65 years, and relative incidences in the 42 days after coadministration of bivalent and influenza vaccines among members of Kaiser Permanente Southern California during the period from September 1, 2022, to March 31, 2023.

	Number of events, n				Relative incidence (95% CI)
	Risk interval	Control interval	NBRs^b^		
Overall	10	14	835	2.34 (0.97-5.65)	
With history of SARS-CoV-2^c^	4	3	190	4.27 (0.97-18.85)	
Without history of SARS-CoV-2	6	11	645	1.76 (0.57-5.45)	

^a^Confirmation by chart review.

^b^NBR: nonbivalent recipient. These were eligible individuals who did not receive a bivalent vaccine but had completed a primary series of COVID-19 vaccination and had their last monovalent dose ≥60 days before September 1, 2022. Inclusion of these events helps to adjust for temporal trends (seasonality). A confirmation rate of 65% was applied to ischemic stroke events among NBR.

^c^Had SARS-CoV-2 infection (ie, SARS-CoV-2 positive laboratory test or a COVID-19 diagnosis) during the year prior (August 31, 2021-August 31, 2022).

### Risk of Ischemic Stroke Following the Moderna Bivalent COVID-19 Vaccine

When using a risk interval of 21 days following Moderna bivalent vaccination, the overall risk of ischemic stroke was not elevated from the analysis of electronically identified ischemic stroke events (RI=0.91, 95% CI 0.71-1.15), a finding that held true across all subgroup analyses by age, coadministration of influenza vaccine, and history of SARS-CoV-2 infection (Multimedia Appendix 4). However, extending the risk interval to 42 days after Moderna bivalent vaccination showed an increased risk of ischemic stroke among individuals younger than 65 years of age who had a documented history of SARS-CoV-2 infection, with an RI of 2.62 (95% CI 1.13-6.03). This subgroup involved a total of 36 ischemic stroke events among recipients of the Moderna bivalent COVID-19 vaccine (Table 4).

Of the 36 ischemic stroke events, 1 was a hemorrhagic stroke and 12 were not confirmed as true events through medical chart review, yielding a confirmation rate of 64% (n=23). After using a risk interval of 1-42 days following the Moderna bivalent vaccination and applying a 64% confirmation rate to ischemic stroke events among NBR, the RI derived from analyzing confirmed ischemic stroke events among those aged younger than 65 years who had a documented history of SARS-CoV-2 infection was 2.24 (95% CI 0.78-6.47; *P*=.14).

**Table 4:** Numbers of electronically identified ischemic stroke events and relative incidences in the 42 days after Moderna bivalent COVID-19 vaccination among members of Kaiser Permanente Southern California during the period from September 1, 2022, to March 31, 2023.

		All ages						<65 years old						≥65 years old				
		Number of events, n				Relative incidence (95% CI)		Number of events, n				Relative incidence (95% CI)		Number of events, n				Relative incidence (95% CI)
		Risk interval	Control interval	NBRs^a^			Risk interval		Control interval	NBRs			Risk interval		Control interval	NBRs		
**All recipients**																		
	Overall	161	666	3049	0.92 (0.76-1.12)		34		158	1278	0.99 (0.64-1.53)		127		508	1771	0.90 (0.72-1.13)	
	With history of SARS-CoV-2^b^	28	111	565	1.02 (0.66-1.59)		10		26	292	2.62 (1.13-6.03)		18		85	273	0.71 (0.42-1.19)	
	Without history of SARS-CoV-2	133	555	2484	0.90 (0.72-1.12)		24		132	986	0.73 (0.43-1.23)		109		423	1498	0.95 (0.74-1.21)	
**Coadministration of influenza vaccine, overall**																		
	Overall	19	72	3049	0.80 (0.45-1.43)		8		20	1278	1.42 (0.59-3.43)		11		52	1771	0.60 (0.28-1.29)	
	With history of SARS-CoV-2^b^	3	14	565	0.59 (0.15-2.30)		3		4	292	2.43 (0.54-10.87)		0		10	273	N/A^c^	
	Without history of SARS-CoV-2	16	58	2484	0.85 (0.45-1.63)		5		16	986	1.05 (0.36-3.08)		11		42	1498	0.80 (0.36-1.76)	
**No coadministration of influenza vaccine, overall**																		
	Overall	142	594	3049	0.94 (0.76-1.16)		26		138	1278	0.89 (0.54-1.47)		116		456	1771	0.95 (0.75-1.19)	
	With history of SARS-CoV-2^b^	25	97	565	1.11 (0.69-1.76)		7		22	292	2.69 (0.98-7.36)		18		75	273	0.83 (0.49-1.42)	
	Without history of SARS-CoV-2	117	497	2484	0.91 (0.71-1.15)		19		116	986	0.67 (0.37-1.21)		98		381	1498	0.97 (0.75-1.26)	

^a^NBR: nonbivalent recipient. These were eligible individuals who did not receive a bivalent vaccine but had completed a primary series of COVID-19 vaccination and had their last monovalent dose ≥60 days before September 1, 2022. Inclusion of these events helps to adjust for temporal trends (seasonality). The same NBR population was used in overall bivalent analyses, as well as bivalent analyses stratified by coadministration of influenza vaccine.

^b^Had SARS-CoV-2 infection (ie, SARS-CoV-2 positive laboratory test or a COVID-19 diagnosis) during the year prior (August 31, 2021-August 31, 2022).

^c^N/A: not applicable.

### Sex-Stratified Analyses

Further analyses were conducted to examine if the risk of ischemic stroke after bivalent vaccination differed by sex for those analyses indicating a potential increase in risk of ischemic stroke (Multimedia Appendix 5). The risk of ischemic stroke was not significantly increased after bivalent vaccination in the sex-stratified analyses. Due to limited sample sizes, the CIs of RIs were wide. The SAS and R codes are available for preparing data and fitting the event-dependent SCCS models (Multimedia Appendix 6).

## Discussion

### Principal Findings

These SCCS analyses did not find evidence that the risk of ischemic stroke was elevated during the 1-21–day postvaccination risk interval in both overall and subgroup analyses by age (younger than 65 years vs 65 years or older), prior history of SARS-CoV-2 infection, and coadministration of influenza vaccine, for both Pfizer-BioNTech and Moderna bivalent vaccines. However, based on electronically identified events, the risk of ischemic stroke was increased within the 1-42–day window after vaccination among those aged younger than 65 years who received their Pfizer-BioNTech bivalent vaccine and influenza vaccine on the same day; the risk was even higher among those who also had a documented SARS-CoV-2 infection history. After conducting a chart review of ischemic stroke events, the point estimate for the risk of ischemic stroke was still elevated in a risk interval of 1-42 days for these 2 subgroup analyses but did not meet the threshold for statistical significance (*P*=.06).

For Moderna bivalent vaccination, an initial increase in the risk of ischemic stroke emerged within the 1-42–day window after vaccination among those aged younger than 65 years who had a documented SARS-CoV-2 infection history. However, after conducting a chart review of ischemic stroke events, the RI was 2.24 but was no longer statistically significantly elevated possibly due to a decreased sample size (*P*=.14).

Our study showed an increased point estimate for the risk of ischemic stroke in a risk interval of 1-42 days only among those aged younger than 65 years who received their Pfizer-BioNTech bivalent vaccine and influenza vaccine on the same day, although not statistically significant. This finding is unique and may be attributed to differences in the study design compared to previous studies. First, our study used a calendar-based observation period spanning from September 1, 2022, to March 31, 2023. This extended timeframe enabled us to use a longer risk window of 1-42 days following vaccination in addition to the risk interval of 1-21 days in previous studies. Second, we did not exclude individuals with a history of ischemic stroke, but we did apply criteria to increase the likelihood that ischemic stroke events during the study period represented a new ischemic stroke episode. Nevertheless, it is possible that there was interaction between bivalent vaccination and a history of ischemic stroke. Furthermore, in subgroup analyses, we considered the influence of the history of SARS-CoV-2 infection. SARS-CoV-2 infection is associated with an increased risk of ischemic stroke [28,29] and risk factors for SARS-CoV-2 infection may overlap with risk factors for ischemic stroke. There is a potential interaction between bivalent vaccination and a history of SARS-CoV-2 infection. The finding that the point estimate for the risk of ischemic stroke was elevated among individuals aged younger than 65 years but not among individuals aged 65 years or older is also biologically plausible. This may be due to the relatively heightened immune response and subsequent inflammation in the younger age group versus the older age group, and the fact that inflammation has been shown to be associated with an increased risk of ischemic stroke [30,31]. Moreover, a smaller proportion of younger adults opted for bivalent vaccination [32], and those who did might have had a higher prevalence of comorbidities or poorer overall health status.

### Limitations and Strengths

This study had several limitations. First, the study took place in a single health care system. Additionally, the number of ischemic stroke events in individuals with a documented SARS-CoV-2 infection history who received coadministration of Pfizer-BioNTech bivalent vaccine and influenza vaccine was very small. This raises concerns about the validity of the asymptotic large sample assumptions that underlie both the 95% CIs and *P* values. Second, we did not conduct a chart review of ischemic stroke events among NBR; these events contributed to establishing baseline rates of ischemic stroke events during the study period. In addressing this issue, when analyzing chart-confirmed ischemic stroke events among recipients of bivalent vaccine, we applied the confirmation rate of ischemic stroke events among recipients of bivalent vaccine to ischemic stroke events among NBR. Moreover, we also did not undertake a chart review of ischemic stroke events from those analyses when safety signals were absent. Third, while we excluded ischemic stroke events occurring within 30 days of SARS-CoV-2 infection, it is possible that some ischemic stroke events included in the analyses involved individuals with asymptomatic or mild COVID-19 disease who did not have a documented SARS-CoV-2 infection. Fourth, the elevated point estimate for the risk of ischemic stroke, while not statistically significant, was observed within the 1-42–day period following the coadministration of the Pfizer-BioNTech bivalent vaccine and influenza vaccine. This risk interval was longer than the 1-21 days or 1-28 days investigated in earlier research [10,13,33]. However, the biological plausibility for the occurrence of a vaccine-related ischemic stroke beyond 28 days remains uncertain. Fifth, unaccounted time-varying confounders could have also influenced the findings. Finally, our analysis did not adjust for multiple subgroup analyses by age, coadministration of bivalent COVID-19 vaccine and influenza vaccine, and history of SARS-CoV-2 infection. These specific subgroup analyses were prespecified due to their potential safety concerns. The decision not to make multiple comparison adjustments was deliberate, aimed at ensuring that any potential vaccine safety concern could be detected.

The study also had several strengths. First, we addressed the impact of previous ischemic stroke events on bivalent COVID-19 vaccination by using an event-dependent modified SCCS design. Second, we explored effect heterogeneity by conducting subgroup analyses based on factors such as age, documented history of SARS-CoV-2 infection, and coadministration of influenza vaccine. Third, to adjust for temporal trends, we included ischemic stroke events among NBR. This strategy not only enhanced the accuracy of estimating the baseline rate but also improved the statistical power for identifying potential safety signals. Finally, we reanalyzed ischemic stroke events that were confirmed through chart review for those analyses where safety signals were detected.

Future research should include several key aspects to further enhance the validity and robustness of our findings. Collaborative efforts with additional health care systems will enable us to significantly increase our sample size. A larger sample size could provide sufficient statistical power to conduct sensitivity analyses such as the exclusion of transient ischemic attacks and exclusion of those who had a history of ischemic stroke.

In light of the findings of this study on the risk of ischemic stroke after bivalent COVID-19 vaccination, it is necessary to assess the safety of the monovalent COVID-19 vaccination during 2023-2024 for several reasons. First, while the bivalent COVID-19 vaccines included 2 components (BA.4 and BA.5), the monovalent COVID-19 vaccines during the 2023-2024 season included 1 component (XBB.1.5). This change in vaccine composition warrants continued surveillance to assess any differential safety profiles. Second, during the 2023-2024 season, coadministration of monovalent COVID-19 vaccine and influenza vaccine on the same day was possible given the timing of availability of both products and the recommendation by the CDC.

### Conclusions

We found no evidence to suggest that the Pfizer-BioNTech bivalent vaccine increased the risk of ischemic stroke among individuals aged older than 65 years, consistent with the attenuated signal from the VSD surveillance that motivated this study. We found an elevated point estimate for the risk of ischemic stroke within 1-42 days (but not within 1-21 days) after the coadministration of the Pfizer-BioNTech bivalent vaccine and influenza vaccine among individuals younger than 65 years old that did not reach statistical significance, although the sample size was limited. Future studies with a larger sample size are needed to evaluate the association between bivalent COVID-19 vaccination and ischemic stroke, as well as contributing factors such as the history of SARS-CoV-2 infection. Any potential risks of ischemic stroke associated with bivalent COVID-19 vaccination must be balanced against the potential benefits of bivalent COVID-19 vaccination in preventing COVID-19–associated ischemic stroke and severe COVID-19 disease.

## Appendix

Multimedia Appendix 1 Definition of electronically identified ischemic strokes.

Multimedia Appendix 2 Descriptive statistics of Charlson Comorbidity Index by vaccine type and age (<65 years old and ≥65 years old) among members of Kaiser Permanente Southern California who had ischemic stroke events during the period from September 1, 2022, to March 31, 2023.

Multimedia Appendix 3 Numbers of electronically identified ischemic stroke events and relative incidences in the 21 days after Pfizer-BioNTech bivalent COVID-19 vaccination among members of Kaiser Permanente Southern California during the period from September 1, 2022, to March 31, 2023.

Multimedia Appendix 4 Numbers of electronically identified ischemic stroke events and relative incidences in the 21 days after Moderna bivalent COVID-19 vaccination among members of Kaiser Permanente Southern California during the period from September 1, 2022, to March 31, 2023.

Multimedia Appendix 5 Stratified analyses by sex for those analyses that signaled using electronically identified ischemic stroke events: numbers of confirmed ischemic stroke events and relative incidences in the 42 days after bivalent vaccination among members of Kaiser Permanente Southern California aged <65 years during the period from September 1, 2022, to March 31, 2023.

Multimedia Appendix 6 SAS and R codes for preparing data and fitting the dependent SCCS models.

## Abbreviations

AR: attributable risk
CDC: US Centers for Disease Control and Prevention
FDA: US Food and Drug Administration
ICD-10: International Classification of Diseases, Tenth Revision
KPSC: Kaiser Permanente Southern California
mRNA: messenger ribonucleic acid
NBR: nonbivalent recipient
NNH: number needed to harm
RI: relative incidence
SCCS: self-controlled case series
VAERS: Vaccine Adverse Event Reporting System
VSD: Vaccine Safety Datalink

## Data Availability

The data sets generated and analyzed during this study are not publicly available due to potentially identifying or sensitive patient information but are available from the corresponding author on reasonable request.
