# Supplementary material for: Ischemic Stroke After Bivalent COVID-19 Vaccination: Self-Controlled Case Series Study
Source: JMIR Public Health Surveill. 2024 Jun 25;10:e53807. doi: 10.2196/53807 (PMC11234065; doi:10.2196/53807)
Supplement: Multimedia Appendix 1 [file publichealth_v10i1e53807_app1.docx]

| **Incident ischemic stroke in emergency department or inpatient settings** | | **Adjust ischemic stroke onset if the ICD-10 code occurred in the 1 day prior to the incident case in all settings** | | **Exclude ischemic stroke cases due to other possible causes if the ICD-10 code occurred in the time period noted prior to the incident case (not including the same day) in all settings** | | **Exclude ischemic stroke cases due to other possible causes if the ICD-10 code occurred on the same day as the incident case** | |
| --- | --- | --- | --- | --- | --- | --- | --- |
| ICD-10 | Description | ICD-10 | Description | ICD-10 | Description | ICD-10 | Description |
| G45.8 | Other transient cerebral ischemic attacks and related syndromes | Z92.82 | Status post administration of tPA (rtPA) in a different facility within the last 24 hours prior to admission to current facility | I48.* | Atrial fibrillation and flutter (if seen ever prior to the incident case) | S15.* | Injury of blood vessels at neck level |
| G45.9 | Transient cerebral ischemic attack, unspecified | R51.* | Headache | I21.* | Acute myocardial infarction  (if seen within 28 days prior to the incident case) | I74.* | Arterial embolism and thrombosis |
| I63.* | Cerebral infarction | R47.* | Speech disturbances, not elsewhere classified | S15.* | Injury of blood vessels at neck level (if seen within 1 day prior to the incident case) | Physical trauma codes - see Footnote A | |
|  |  | R29.810 | Facial weakness | I74.* | Arterial embolism and thrombosis (if seen within 1 day prior to the incident case) |  |  |
|  |  | R53.1 | Weakness | D57.* | Sickle-cell disorders (if seen EVER prior to the incident case) |  |  |
|  |  | R42.* | Dizziness and giddiness | D68.5* | Primary thrombophilia (if seen EVER prior to the incident case) |  |  |
|  |  | R41.82 | Altered mental status, unspecified | Exclude if SARS-CoV2 infection^$^ occurred in the last 30 days prior to the incident case (not including same day) | |  |  |
|  |  | R40.4 | Transient alternation of awareness |  |  |  |  |
|  |  | G81.9* | Hemiplegia, unspecified |  |  |  |  |
|  |  | H53.9 | Unspecified visual disturbance |  |  |  |  |
|  |  | H53.13* | Sudden visual loss |  |  |  |  |

*=any remaining characters; ^$^SARS-CoV-2 positive laboratory test or a COVID-19 diagnosis.

| **Footnote A: Physical Trauma Codes** | |
| --- | --- |
| Trauma - external causes of morbidity (V00-Y99): | |
| V00-V09 | Pedestrian injured in transport accident |
| V10-V19 | Pedal cycle rider injured in transport accident |
| V20-V29 | Motorcycle rider injured in transport accident |
| V30-V39 | Occupant of three-wheeled motor vehicle injured in transport accident |
| V40-V49 | Car occupant injured in transport accident |
| V50-V59 | Occupant of pick-up truck or van injured in transport accident |
| V60-V69 | Occupant of heavy transport vehicle injured in transport accident |
| V70-V79 | Bus occupant injured in transport accident |
| V80-V89 | Other land transport accidents |
| V90-V94 | Water transport accidents |
| V95-V97 | Air and space transport accidents |
| V98-V99 | Other and unspecified transport accidents |
| W00-W19 | Slipping, tripping, stumbling and falls |
| W20-W49 | Exposure to inanimate mechanical forces |
| W50-W64 | Exposure to animate mechanical forces |
| W65-W74 | Accidental non-transport drowning and submersion |
| W85-W99 | Exposure to electric current, radiation and extreme ambient air temperature and pressure |
| X00-X08 | Exposure to smoke, fire and flames |
| X10-X19 | Contact with heat and hot substances |
| X30-X39 | Exposure to forces of nature |
| X50.* | Overexertion and strenuous or repetitive movements |
| X52-X58 | Accidental exposure to other specified factors |
| X71-X83 | Intentional self-harm |
| X92-Y09 | Assault |
| Y21-Y33 | Event of undetermined intent |
| Y35-Y38 | Legal intervention, operations of war, military operations, and terrorism |
| Y62-Y69 | Misadventures to patients during surgical and medical care |
| Y70-Y82 | Medical devices associated with adverse incidents in diagnostic and therapeutic use |
| Y83-Y84 | Surgical and other medical procedures as the cause of abnormal reaction of the patient, or of later complication, without mention of misadventure at the time of the procedure |
| Y90-Y99 | Supplementary factors related to causes of morbidity classified elsewhere |
