# Supplementary material for: Ischemic Stroke After Bivalent COVID-19 Vaccination: Self-Controlled Case Series Study
Source: JMIR Public Health Surveill. 2024 Jun 25;10:e53807. doi: 10.2196/53807 (PMC11234065; doi:10.2196/53807)
Supplement: Multimedia Appendix 2 [file publichealth_v10i1e53807_app2.docx]

|  | Recipients of Pfizer -BioNTech bivalent COVID-19 vaccine (n=1057) | | Recipients of Moderna bivalent COVID-19 vaccine (n=827) | | Non-recipients of bivalent vaccines (n=3049) | |
| --- | --- | --- | --- | --- | --- | --- |
| Charlson Comorbidity Index | <65 years old  (n=278) | ≥65 years old  (n=779) | <65 years old  (n=192) | ≥65 years old  (n=635) | <65 years old  (n=1278) | ≥65 years old  (n=1771) |
| 0 | 46.4% | 17.5% | 43.8% | 16.5% | 50.0% | 15.8% |
| 1 | 20.1% | 18.4% | 16.7% | 15.6% | 20.7% | 14.7% |
| 2 | 11.9% | 17.3% | 12.5% | 16.2% | 11.4% | 14.0% |
| 3+ | 21.6% | 46.8% | 27.0% | 51.7% | 17.9% | 55.5% |
| Median (IQR) | 1 (0–2) | 2 (1–5) | 1 (0–3) | 3 (1–5) | 0.5 (0–2) | 3 (1–5) |

IQR= interquartile range
