# Supplementary material for: Ischemic Stroke After Bivalent COVID-19 Vaccination: Self-Controlled Case Series Study
Source: JMIR Public Health Surveill. 2024 Jun 25;10:e53807. doi: 10.2196/53807 (PMC11234065; doi:10.2196/53807)
Supplement: Multimedia Appendix 3 [file publichealth_v10i1e53807_app3.docx]

|  | **All ages** | | | | **<65 years old** | | | | **≥65 years old** | | | |
| --- | --- | --- | --- | --- | --- | --- | --- | --- | --- | --- | --- | --- |
|  | Number of events | | |  | Number of events | | |  | Number of events | | |  |
|  | Risk interval | Control interval | NBR^§^ | Relative incidence (95% CI) | Risk interval | Control interval | NBR^§^ | Relative incidence (95% CI) | Risk interval | Control interval | NBR^§^ | Relative incidence (95% CI) |
| **Overall** | 103 | 954 | 3049 | 0.90 (0.73–1.12) | 19 | 259 | 1278 | 0.68 (0.42–1.12) | 84 | 695 | 1771 | 0.98 (0.77–1.25) |
| With history of SARS-CoV-2^₽^ | 12 | 162 | 565 | 0.68 (0.39–1.19) | 2 | 66 | 292 | 0.52 (0.18–1.51) | 10 | 96 | 273 | 0.77 (0.40–1.47) |
| Without history of SARS-CoV-2 | 91 | 792 | 2484 | 0.96 (0.76–1.21) | 17 | 193 | 986 | 0.75 (0.43–1.32) | 74 | 599 | 1498 | 1.02 (0.79–1.33) |
| **Co-administration of influenza vaccine, overall** | 22 | 145 | 3049 | 1.01 (0.64–1.58) | 6 | 31 | 1278 | 1.38 (0.59–3.22) | 16 | 114 | 1771 | 0.92 (0.53–1.58) |
| With history of SARS-CoV-2^₽^ | 3 | 26 | 565 | 0.80 (0.30–2.17) | 1 | 9 | 292 | 1.35 (0.33–5.52) | 2 | 17 | 273 | 0.55 (0.14–2.12) |
| Without history of SARS-CoV-2 | 19 | 119 | 2484 | 1.06 (0.64–1.77) | 5 | 22 | 986 | 1.40 (0.49–3.99) | 14 | 97 | 1498 | 1.00 (0.56–1.81) |
| **No co-administration of influenza vaccine, overall** | 81 | 809 | 3049 | 0.87 (0.69–1.12) | 13 | 228 | 1278 | 0.55 (0.30–1.01) | 68 | 581 | 1771 | 0.98 (0.75–1.28) |
| With history of SARS-CoV-2^₽^ | 9 | 136 | 565 | 0.64 (0.33–1.24) | 1 | 57 | 292 | 0.33 (0.07–1.50) | 8 | 79 | 273 | 0.79 (0.38–1.66) |
| Without history of SARS-CoV-2 | 72 | 673 | 2484 | 0.93 (0.72–1.21) | 12 | 171 | 986 | 0.64 (0.33–1.28) | 60 | 502 | 1498 | 1.02 (0.77–1.36) |

^§^Non-bivalent recipients (NBR) were eligible individuals who did not receive a bivalent vaccine but had completed a primary series of COVID-19 vaccination and had their last monovalent dose ≥60 days before 9/1/2022. Inclusion of these events helps to adjust for temporal trends (seasonality). The same NBR population was used in overall bivalent analyses as well as bivalent analyses stratified by co-administration of influenza vaccine. ^₽^ Had SARS-CoV-2 infection (ie, SARS-CoV-2 positive laboratory test or a COVID-19 diagnosis) during the year prior (08/31/2021-08/31/2022).
