# Supplementary material for: Ischemic Stroke After Bivalent COVID-19 Vaccination: Self-Controlled Case Series Study
Source: JMIR Public Health Surveill. 2024 Jun 25;10:e53807. doi: 10.2196/53807 (PMC11234065; doi:10.2196/53807)
Supplement: Multimedia Appendix 4 [file publichealth_v10i1e53807_app4.docx]

|  | **All ages** | | | | **<65 years old** | | | | **≥65 years old** | | | |
| --- | --- | --- | --- | --- | --- | --- | --- | --- | --- | --- | --- | --- |
|  | Number of events | | |  | Number of events | | |  | Number of events | | |  |
|  | Risk interval | Control interval | NBR^§^ | Relative incidence (95% CI) | Risk interval | Control interval | NBR^§^ | Relative incidence (95% CI) | Risk interval | Control interval | NBR^§^ | Relative incidence (95% CI) |
| **Overall** | 82 | 745 | 3049 | 0.91 (0.71–1.15) | 13 | 179 | 1278 | 0.63 (0.35–1.14) | 69 | 566 | 1771 | 0.99 (0.76–1.29) |
| With history of SARS-CoV-2^₽^ | 10 | 129 | 565 | 0.60 (0.32–1.14) | 3 | 33 | 292 | 0.87 (0.25–2.97) | 7 | 96 | 273 | 0.52 (0.25–1.10) |
| Without history of SARS-CoV-2 | 72 | 616 | 2484 | 0.98 (0.75–1.28) | 10 | 146 | 986 | 0.58 (0.30–1.13) | 62 | 470 | 1498 | 1.11 (0.83–1.47) |
| **Co-administration of influenza vaccine, overall** | 7 | 84 | 3049 | 0.53 (0.23–1.21) | 4 | 24 | 1278 | 1.15 (0.39–3.40) | 3 | 60 | 1771 | 0.32 (0.09–1.13) |
| With history of SARS-CoV-2^₽^ | 1 | 16 | 565 | 0.39 (0.05–3.06) | 1 | 6 | 292 | 1.03 (0.12–8.79) | 0 | 10 | 273 | N/A |
| Without history of SARS-CoV-2 | 6 | 68 | 2484 | 0.56 (0.23–1.39) | 3 | 18 | 986 | 1.14 (0.33–3.93) | 3 | 50 | 1498 | 0.39 (0.10–1.42) |
| **No co-administration of influenza vaccine, overall** | 75 | 661 | 3049 | 0.96 (0.75–1.24) | 9 | 155 | 1278 | 0.53 (0.26–1.06) | 66 | 506 | 1771 | 1.09 (0.83–1.43) |
| With history of SARS-CoV-2^₽^ | 9 | 113 | 565 | 0.63 (0.32–1.23) | 2 | 27 | 292 | 0.79 (0.18–3.54) | 7 | 86 | 273 | 0.58 (0.27–1.23) |
| Without history of SARS-CoV-2 | 66 | 548 | 2484 | 1.04 (0.79–1.37) | 7 | 128 | 986 | 0.48 (0.22–1.06) | 59 | 420 | 1498 | 1.22 (0.91–1.64) |

^§^Non-bivalent recipients (NBR) were eligible individuals who did not receive a bivalent vaccine but had completed a primary series of COVID-19 vaccination and had their last monovalent dose ≥60 days before 9/1/2022. Inclusion of these events helps to adjust for temporal trends (seasonality). The same NBR population was used in overall bivalent analyses as well as bivalent analyses stratified by co-administration of influenza vaccine. ^₽^ Had SARS-CoV-2 infection (ie, SARS-CoV-2 positive laboratory test or a COVID-19 diagnosis) during the year prior (08/31/2021-08/31/2022).
