# Supplementary material for: Ischemic Stroke After Bivalent COVID-19 Vaccination: Self-Controlled Case Series Study
Source: JMIR Public Health Surveill. 2024 Jun 25;10:e53807. doi: 10.2196/53807 (PMC11234065; doi:10.2196/53807)
Supplement: Multimedia Appendix 5 [file publichealth_v10i1e53807_app5.docx]

| **Pfizer bivalent vaccine** | Female | | | | Male | | | |
| --- | --- | --- | --- | --- | --- | --- | --- | --- |
|  | Number of events | | |  | Number of events | | |  |
|  | Risk interval | Control interval | NBR^§^ | Relative incidence (95% CI) | Risk interval | Control interval | NBR^§^ | Relative incidence (95% CI) |
| Co-administration of influenza vaccine, overall | 5 | 7 | 417 | 2.43 (0.72–8.16) | 5 | 7 | 418 | 2.22 (0.67–7.34) |
| With history of SARS-CoV-2^₽^ | 3 | 2 | 113 | 5.22 (0.85–32.01) | 1 | 1 | 80 | 2.51 (0.22–29.17) |
| Without history of SARS-CoV-2 | 2 | 5 | 304 | 1.36 (0.23–7.94) | 4 | 6 | 338 | 2.19 (0.50–9.60) |
| **Moderna bivalent vaccine** | Female | | | | Male | | | |
|  | Number of events | | |  | Number of events | | |  |
|  | Risk interval | Control interval | NBR^£^ | Relative incidence (95% CI) | Risk interval | Control interval | NBR^£^ | Relative incidence (95% CI) |
| With history of SARS-CoV-2^₽^ | 4 | 8 | 106 | 3.78 (0.69–20.68) | 3 | 8 | 78 | 1.61 (0.37–7.07) |

^€^Confirmation by chart review. ^§^Non-bivalent recipients (NBR) were eligible individuals who did not receive a bivalent vaccine but had completed a primary series of COVID-19 vaccination and had their last monovalent dose ≥60 days before 9/1/2022. Inclusion of these events helps to adjust for temporal trends (seasonality). A confirmation rate of 65% was applied to ischemic stroke events among NBR. ^₽^ Had SARS-CoV-2 infection (ie, SARS-CoV-2 positive laboratory test or a COVID-19 diagnosis) during the year prior (08/31/2021-08/31/2022). ^£^Non-bivalent recipients (NBR) were eligible individuals who did not receive a bivalent vaccine but had completed a primary series of COVID-19 vaccination and had their last monovalent dose ≥60 days before 9/1/2022. Inclusion of these events helps to adjust for temporal trends (seasonality). A confirmation rate of 64% was applied to ischemic stroke events among NBR.
