# Supplementary material for: Ischemic Stroke After Bivalent COVID-19 Vaccination: Self-Controlled Case Series Study
Source: JMIR Public Health Surveill. 2024 Jun 25;10:e53807. doi: 10.2196/53807 (PMC11234065; doi:10.2196/53807)
Supplement: Multimedia Appendix 6 [file publichealth_v10i1e53807_app6.docx]

**SAS and R codes for preparing data and fitting the dependent SCCS models**

/***********************************************************************************************************

*Step 1: prepare data, all days were anchored at the start of the study period: 9/1/2022

*the original dataset pfizer_overall has the following variables: mrn, medical record number

*vac_bivalent1, the date when an individual received the first bivalent vaccination

*admitdate, the date when an individual had medical encounters with an ischemic stroke DX

*fup_end, the date when follow-up ended,the earliest of death, disenrollment, end of study,

*receiving the 2nd bivalent vaccination

*********************************************************************************************************/

data pfizer_overall_4SCCS;

set pfizer_overall;

VAC1_day=vac_bivalent1-mdy(**9**,**1**,**2022**)+**1**;

is_day=admitdate-mdy(**9**,**1**,**2022**)+**1**;

case=mrn***1**;

fu_start1=**1**;

fu_end1=fup_end-mdy(**9**,**1**,**2022**)+**1**;

**run**;

* co-adm with flu shots under 65 years old;

**data** pfizer_coadm_under65;

set pfizer_overall_4SCCS;

**delete those that were reviewed and were not true case;

if chart_review=**1** and confirmation='N' then delete;

if age<**65**;

if vac_bivalent1=**.** then output pfizer_coadm_under65; *unvaccinated cases;

else if flu_vacdate ne **.** and (flu_vacdate=vac_bivalent1) then output pfizer_coadm_under65; **co-adm cases;

**run**;

*Step 2: random sampling (RS) 68% unexposed cases: five samples;

**%macro** randsamp(rs);

data pfizer_coadm_under65_rs&rs;

length sample $ **10**;

seed=**42134**+&rs;

set pfizer_coadm_under65;

if chart_review=**1** and confirmation='Y' then do;

rs=**1**;

sample='vac';

end;

else do;

rs=ranbin(seed,**1**,**0.68**);

sample='unvac';

end;

if rs=**1**;

run;

**%mend**;

%***randsamp***(rs=**1**); %***randsamp***(rs=**2**); %***randsamp***(rs=**3**);%***randsamp***(rs=**4**);%***randsamp***(rs=**5**);

*Step 3: export these five SAS datasets into five Tab delimited file (*.txt);

*Step 4: conduct dependent SCCS analyses using SCCS R package with risk interval 1-42 days after vaccination;

library(SCCS)

seas<- cumsum(c(**30**,**31**,**30**,**31**,**31**,**28**,**31**))

#read in data

setwd ("your folder where Tab delimited files were saved ")

pfizer_coadm_under65_rs1 <- read.table("pfizer_coadm_under65_rs1.txt", sep="\t", header=TRUE)

pfizer_coadm_under65_rs1

is.pfizer_coadm_under65_rs1_rw42 <- eventdepenexp(indiv=case, astart=fu_start1, aend=fu_end1,

aevent=is_day, adrug=VAC1_day,

aedrug=VAC1_day+**42**,

sameexpopar=F, agegrp=seas,

data=pfizer_coadm_under65_rs1)

is.pfizer_coadm_under65_rs1_rw42

pfizer_coadm_under65_rs2 <- read.table("pfizer_coadm_under65_rs2.txt", sep="\t", header=TRUE)

pfizer_coadm_under65_rs2

is.pfizer_coadm_under65_rs2_rw42 <- eventdepenexp(indiv=case, astart=fu_start1, aend=fu_end1,

aevent=is_day, adrug=VAC1_day,

aedrug=VAC1_day+**42**,

sameexpopar=F, agegrp=seas,

data=pfizer_coadm_under65_rs2)

is.pfizer_coadm_under65_rs2_rw42

pfizer_coadm_under65_rs3 <- read.table("pfizer_coadm_under65_rs3.txt", sep="\t", header=TRUE)

pfizer_coadm_under65_rs3

is.pfizer_coadm_under65_rs3_rw42 <- eventdepenexp(indiv=case, astart=fu_start1, aend=fu_end1,

aevent=is_day, adrug=VAC1_day,

aedrug=VAC1_day+**42**,

sameexpopar=F, agegrp=seas,

data=pfizer_coadm_under65_rs3)

is.pfizer_coadm_under65_rs3_rw42

pfizer_coadm_under65_rs4 <- read.table("pfizer_coadm_under65_rs4.txt", sep="\t", header=TRUE)

pfizer_coadm_under65_rs4

is.pfizer_coadm_under65_rs4_rw42 <- eventdepenexp(indiv=case, astart=fu_start1, aend=fu_end1,

aevent=is_day, adrug=VAC1_day,

aedrug=VAC1_day+**42**,

sameexpopar=F, agegrp=seas,

data=pfizer_coadm_under65_rs4)

is.pfizer_coadm_under65_rs4_rw42

pfizer_coadm_under65_rs5 <- read.table("pfizer_coadm_under65_rs5.txt", sep="\t", header=TRUE)

pfizer_coadm_under65_rs5

is.pfizer_coadm_under65_rs5_rw42 <- eventdepenexp(indiv=case, astart=fu_start1, aend=fu_end1,

aevent=is_day, adrug=VAC1_day,

aedrug=VAC1_day+**42**,

sameexpopar=F, agegrp=seas,

data=pfizer_coadm_under65_rs5)

is.pfizer_coadm_under65_rs5_rw42

*Step **5**: pool estimates across results from 5 random samples;

**results from R were input into the following dataset;

**data** pf_coadm_lt65_results;

length parameter $ **15**;

input parameter Estimate exp_beta StdErr;

cards;

VAC1_day1 0.83201 2.297935 0.4464640

VAC1_day1 0.86362 2.371724 0.4459690

VAC1_day1 0.91829 2.50500 0.44667

VAC1_day1 0.81080 2.24970 0.44419

VAC1_day1 0.84373 2.32502 0.44618

;

**data** pf_coadm_lt65_results1;

drop parameter Estimate exp_beta StdErr;

set pf_coadm_lt65_results;

beta=estimate;

beta_stderr=stderr;

_imputation_=_n_;

**run**;

**proc** **mianalyze** data=pf_coadm_lt65_results1;

modeleffects beta;

stderr beta_stderr;

ods output parameterestimates=pf_coadm_lt65_pool;

**run**;

**data** pf_coadm_lt65_pool_RI;

set pf_coadm_lt65_pool;

RI=round(**100***exp(Estimate))/**100**;

RI_lower_CI=round(**100***exp(LCLMean))/**100**;

RI_upper_CI=round(**100***exp(UCLMean))/**100**;

**run**;

**proc** **print** data=pf_coadm_lt65_pool_RI;

var RI RI_lower_CI RI_upper_CI;

**run**;
